# Initiating PeriCBD to probe perinatal influences on neurodevelopment during 3–10 years in China

**DOI:** 10.1038/s41597-024-03211-5

**Published:** 2024-05-07

**Authors:** Yin-Shan Wang, Xue-Ting Su, Li Ke, Qing-Hua He, Da Chang, JingJing Nie, XinLi Luo, Fumei Chen, Jihong Xu, Cai Zhang, Shudong Zhang, Shuyue Zhang, Huiping An, Rui Guo, Suping Yue, Wen Duan, Shichao Jia, Sijia Yang, Yankun Yu, Yang Zhao, Yang Zhou, Li-Zhen Chen, Xue-Ru Fan, Peng Gao, Chenyu Lv, Ziyun Wu, Yunyan Zhao, Xi Quan, Feng Zhao, Yanchao Mu, Yu Yan, Wenchao Xu, Jie Liu, Lixia Xing, Xiaoqin Chen, Xiang Wu, Lanfeng Zhao, Zhijuan Huang, Yanzhou Ren, Hongyan Hao, Hui Li, Jing Wang, Qing Dong, Liyan Chen, Ruiwang Huang, Siman Liu, Yun Wang, Qi Dong, Xi-Nian Zuo

**Affiliations:** 1 State Key Laboratory of Cognitive Neuroscience and Learning, Beijing Normal University, Beijing, 100875 China; 2 Developmental Population Neuroscience Research Center, International Data Group/McGovern Institute for Brain Research, Beijing Normal University, Beijing, 100875 China; 3 Department of Military Operational Medical Protection, Chinese PLA Center for Disease Control and Prevention, Beijing, 100850 China; 4 Collaborative Innovation Center of Assessment for Basic Education Quality, Beijing Normal University, Beijing, 100875 China; 5 Faculty of Psychology, Southwest University, Chongqing, 400715 China; 6 National Research Institute for Health Commission, Beijing, 100081 China; 7 Faculty of Education, Beijing Normal University, Beijing, 100875 China; 8 Department of Psychology, Faculty of Education, Guangxi Normal University, Guilin, 541001 China; 9 Anyang Maternal and Child Health Care Hospital, Anyang, 455000 China; 10 People’s Hospital of Liangping District, Chongqing, 405200 China; 11 Anyang Preschool Education College, Anyang, 456150 China; 12 College of Information and Computer, Taiyuan University of Technology, Taiyuan, 030024 China; 13 School of Psychology, South China Normal University, Guangzhou, 510631 China; 14 School of Humanities and Social Sciences, Beijing Institute of Technology, Beijing, 100081 China

**Keywords:** Cognitive neuroscience, Human behaviour

## Abstract

Adverse perinatal factors can interfere with the normal development of the brain, potentially resulting in long-term effects on the comprehensive development of children. Presently, the understanding of cognitive and neurodevelopmental processes under conditions of adverse perinatal factors is substantially limited. There is a critical need for an open resource that integrates various perinatal factors with the development of the brain and mental health to facilitate a deeper understanding of these developmental trajectories. In this Data Descriptor, we introduce a multicenter database containing information on perinatal factors that can potentially influence children’s brain-mind development, namely, periCBD, that combines neuroimaging and behavioural phenotypes with perinatal factors at county/region/central district hospitals. PeriCBD was designed to establish a platform for the investigation of individual differences in brain-mind development associated with perinatal factors among children aged 3–10 years. Ultimately, our goal is to help understand how different adverse perinatal factors specifically impact cognitive development and neurodevelopment. Herein, we provide a systematic overview of the data acquisition/cleaning/quality control/sharing, processes of periCBD.

## Background & Summary

Adverse perinatal factors, including preterm birth, low birth weight, and other adverse conditions, are the leading causes of neonatal mortality and are associated with long-term physical, neurodevelopmental, and socioeconomic effects. The perinatal period refers to the time from 22 gestational weeks to one week post birth. It is a critical phase in foetal and infant brain development characterized by rapid and complex development. Neurodevelopment during this period is crucial and lay the foundation for later cognitive, emotional and behavioural abilities. With the advance of perinatal care, there are increase number of premature babies who survived with no decline risks of long-term disorders, particularly neurologic and developmental disabilities, infants born with adverse perinatal factors are at greater risk for neurodevelopmental disabilities such as mental retardation, cerebral palsy, hearing loss and vision impairments^1,2^. Adverse perinatal factors also associated with difficulties in motor, cognitive, language, and social abilities. There are also evidence shows that adverse perinatal factors are linked with increasing risks of attention-deficit/hyperactivity disorder (ADHD) and autism spectrum disorders (ASD)^3,4^. It is estimated that, 13.4 million infants are born preterm (at < 37 weeks’ gestation) in 2020 which accounting for 9.9% of all births globally^5^. The long-term consequnces of perinatal factors may impact millions of children, their families, and societies. This pose greater burden on both families and societies^6^. Improving the understanding of the cognitive development and neurodevelopment under adverse perinatal factor conditions is of importance to address the burden.

The impact of adverse perinatal factors on neurodevelopment is principally manifested through in brain injury and subsequent abnormalities or delays in developmental functions. Recent advances in neuroimaging techniques, notably magnetic resonance imaging (MRI), have facilitated non-invasive assessment of alterations in brain structure and functionality in adverse perinatal factors children. MRI studies on perinatal infants have disclosed major brain injuries, including white-matter injury, germinal matrix–intraventricular hemorrhage, and cerebellar hemorrhage^7^. In comparison to full-term infants, preterm infants at term-equivalent age (TEA) exhibit discernible differences in cortical surface area and gray matter volume. Parallel findings are observed in diffusion tensor imaging analyses, underscoring the variability of these impacts. Adverse perinatal conditions detrimentally influence specific neurodevelopmental trajectories^8^. Neuroimaging investigations in preterm infants at two and seven years of age demonstrate reduced gray matter volumes in critical regions including the frontal and temporal lobes, cerebellum, and hippocampus, relative to their full-term counterparts^9,10^. The influence of adverse perinatal factors on neurodevelopment exhibits significant heterogeneity; some individuals do not present with complications such as white matter damage or decreased gray matter volume^11^. The degree to which perinatal factors impinge upon neurodevelopment, particularly during early and later childhood, remains an area of ambiguity. The compilation of multimodal imaging data, coupled with phenotype assessments in individuals affected by adverse perinatal factors throughout their perinatal and childhood stages, constitutes a vital data repository for elucidating these issues.

In pursuit of understanding the impact of perinatal factors on neurodevelopment and cognitive growth, the community has established databases encompassing brain imaging and behavioral measurements. For instance, the Evaluation of Preterm Imaging (EPrime) study^12^ and the Victorian Infant Brain Study (VIBeS)^13^ both collect brain imaging and behavioral data pertaining to preterm and low birth weight infants. Moreover, expansive neurodevelopmental databases like the Developing Human Brain Project (dHCP)^14^ and the Growing Up in Singapore Towards healthy Outcomes (GUSTO) birth cohort study^15^ have also amassed perinatal information concurrently with the collection of child data. Nevertheless, to our knowledge, there presently exists no dedicated database that specifically investigates the influence of perinatal factors on the neurodevelopment of Asian children, particularly within the Chinese pediatric population. To further augment the diversity of samples with adverse perinatal factors and to aid in elucidating the impact of perinatal factors on neurodevelopment, we established a comprehensive multicenter brain-behaviour database called Perinatal Factors in Child Brain-mind Development (periCBD). This database encompasses a wide range of evaluations, including brain MRI scans, clinical manifestations of neurodevelopment conditions, and assessments of motor function. During the data collection process, we also considered other factors not associated with neurodevelopment conditions (NDCs), such as intellectual, cognitive, or behavioural problems, to ensure that these factors were appropriately considered and controlled for. This helps us to better isolate and examine the specific influences of perinatal factors on child development. The investigation of perinatal factors, such as maternal age at delivery, duration of pregnancy, and birth weight, was carried out through the collection and analysis of clinical medical data as well as parental questionnaires. At the pilot stage, obstetric and paediatric data were collected from 2011 to 2018, recruitment was conducted at two district hospitals (the Anyang Maternal and Child Health Hospital of Henan Province and the People’s Hospital of Liangping District of Chongqing). Through the establishment of periCBD, we aimed to create a valuable resource that will contribute to a better understanding of the complex relationships among perinatal factors, brain development, and child behaviour.

## Methods

### Overall design

The aim of periCBD is to build a representative, multidimensional brain-behaviour database comprising county-level data on Chinese children aged 3–10 years who experienced perinatal factors. The long-term goal of this project is to create a national resource that covers the 34 provinces in China, with 34 sites strategically distributed across counties and districts. In the initial phase, we selected two pilot sites: the Anyang Maternal and Child Health Hospital of Henan Province (AY site) and the People’s Hospital of Liangping District of Chongqing (LP site). The two sites were chosen to test the effectiveness of our recruitment and data collection methods. Our target population mainly included children at 3–10 years old, as this age stage of development exhibits a higher incidence of NDCs that enables investigation of children with/without perinatal factors and collection of developmental data related to intelligence, motor skills and cognition. By integrating these developmental assessments with neuroimaging data, our aim is to provide a valuable resource for exploring the effects of perinatal factors on brain development. Additionally, we aimed to investigate the associations between of perinatal factors with sensorimotor and cognitive development and the underlying mechanisms involved.

### Recruitment strategy

Tow groups of participants were recruited for the study: the perinatal factor (PF) group and the normal control (NC) group. The PF group consisted of children aged 3–10 years who had experienced adverse perinatal factors, namely preterm birth (gestational age < 37 weeks) and/or low birth weight (birth weight < 2,500 g). To identify eligible individuals, obstetric and paediatric data from 2011 to 2018 were reviewed, and the eligibility of these children was further confirmed through telephone calls. The NC group comprised children without any adverse perinatal factors. They were recruited from local hospitals from June to August 2021. Parents were invited to attend seminars at the hospital, where they were provided with detailed information regarding the objectives and goals of the project. Additionally, they were informed about the scope of the project through posters that were displayed in various local communities. The NC children were selected to match the PF group in terms of age and demographic characteristics. The exclusion criteria were as follows: (a) children with a history of head injury; (b) the Wechsler Preschool and Primary Scale of Intelligence-fourth edition (WPPSI-IV) or the Wechsler Intelligence Scale for Children-fourth edition (WISC-IV) full-scale Intelligence Quotient (FSIQ) standard score lower than 70; (c) Child Behaviour Checklist total problem scale greater than 70. Additionally, children with a gestational or perinatal history of pathologies or risk factors were excluded from the NC group. All the data collected were later double-checked with the information provided by the caregivers of the individual participants to ensure accuracy.

### Participants

**Fig. 1:**
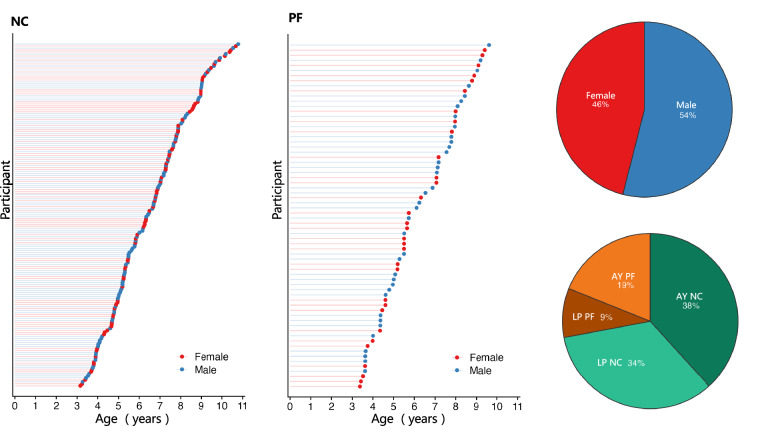
The demographic characteristics of individuals in the periCBD dataset. A total 241 children were involved in this study in the normal control (NC) group and the perinatal factor (PF) group. Among the participants, 46% were female, while 19% of children in the AY group and 9% of children in the LP group had adverse perinatal factors.

A total of 241 children (age: 3–10 years; LP site: 115, AY site: 126) were recruited. There were 68 children in the PF group (AY site: 46; LP site: 22) and 173 children in the NC group (AY site: 80; LP site: 93). Further details about the demographic information are presented in Fig. 1. Both children and their parents/guardians volunteered to participate in data collection and further data sharing, and gave informed consent before participation. This study was approved by the Institutional Review Board (IRB) of the State Key Laboratory of Cognitive Neuroscience and Learning at Beijing Normal University (file number: ICBIR_A_0206_001).

### Data acquisition

#### Demographic, perinatal and family influences

The data collection process involved gathering information on various factors, including maternal age, body mass index (BMI), lifestyle, family socioeconomic status (SES), perinatal risk factors, maternal emotional problems, and parental behaviour. Maternal emotional problems were evaluated using multiple assessment tools, namely, the Edinburgh Postnatal Depression Scale^16^, the Self-Rating Depression Scale^17^, the Trait Depression Scale^18^, the Self-Rating Anxiety Scale^17^, and the State-Trait-Anxiety Inventory^19^. Parental behaviour was evaluated by the Life Event Scale (partial)^20^, Child Neglect Scale (partial)^21^, the Brief Coparenting Relationship Scale (Brief CRS)^22^, and Parenting Sense of Competence Scale (PSOC)^23^. For more details on the applicable age range and duration of these assessments/procedures, please refer to Table 1.

- **Family Socioeconomic Status (SES)** Family socioeconomic status^24^ was assessed by synthesizing five items from three dimensions: parents’ education level, parents’ occupation, and family income. The caregiver provided the answers for this assessment. A higher score indicated a higher family SES. Children’s early family SES was measured by asking participants to indicate their agreement with three statements on a 9-point scale from 1, strongly disagree, to 9, strongly agree: “My family usually had enough money for things when I was growing up,” “I grew up in a relatively wealthy neighborhood,” and “I felt relatively wealthy compared to the other kids in my school.”^25,26^. This scale effectively reflects the quality of the early developmental environment for children. The child’s mother was asked to recall the conditions during pregnancy and the first week of the child’s life. Scores ranged from 1 (strongly disagree) to 5 (strongly agree), with higher scores indicating a higher early family socioeconomic status.
- **Edinburgh Postnatal Depression Scale** The Edinburgh Postnatal Depression Scale is a self-report assessment tool specifically designed to evaluate postnatal depression in mothers. The scale is completed by the mother of the child and consists of 10 items. Each item is rated on a 4-point scale, ranging from 0 to 3. The total score ranges from 0 to 30, with higher scores indicating a higher severity of postnatal depression. When applied to the Chinese sample, the scale demonstrated commendable reliability and validity. It achieved an internal consistency reliability coefficient of 0.76 and a criterion-related validity of 0.584.^16,27^.
- **Self-Rating Depression Scale and Trait Depression Scale** The Self-Rating Depression Scale and the Trait Depression Scale were administered to the child’s mother to assess her current depressive state and trait depression, respectively. The Self-Rating Depression Scale consists of 20 items, which are rated on a scale from 1 to 4. A higher total score indicates a more severe current depression symptoms in the mother. In 2012, Duan Quanquan and Sheng Li conducted a comprehensive investigation into the application of the scale within Chinese clinical settings.^17^. The Trait Depression Scale is a 16-item scale, with items rated on a scale of 1 to 4, and total scores ranging from 16 to 64. A higher total score on this scale reflects a higher level of trait depression in the mother^19^.In the Chinese context, the scale exhibits high reliability, with a coefficient of 0.92 and robust validity (*χ*^2^ = 709.36, df = 103, RMSEA = 0.06, CFI = 0.96, NFI = 0.95, IFI = 0.96)^28^.
- **Self-Rating Anxiety Scale and State-Trait-Anxiety Inventory** The Self-Rating Anxiety Scale and the State-Trait Anxiety Inventory were completed by the mother to assess her current state of anxiety and trait anxiety, respectively. The Self-Rating Anxiety Scale consists of 20 items, each rated on a scale of 1 to 4. A higher total score on this scale indicates more severe anxiety experienced by the mother. Duan Quanquan and Sheng Li investigated the use of the scale in Chinese clinical practice in 2012^17^. The Trait Depression Scale is a 20-item scale with total scores ranging from 20 to 80. Higher scores indicate a higher level of trait anxiety in the mother^19^. In China, the scale exhibits high reliability (0.894) and robust validity (*χ*^2^ = 1571.328, df = 169, RMSEA = 0.061, SRMR = 0.039, CFI = 0.911, TLI = 0.900)^29^.
- **Parental behaviour** To assess various aspects of family dynamics and parental functioning, several scales were employed. First, the Life Event Scale (partial) was used to assess family stress. The caregiver answered 10 items, with higher scores indicating a greater amount of negative life events, i.e., greater perceived family stress. The Life Event Scale has good internal consistency reliability (0.90) and acceptable levels of construct validity (*χ*^2^/*df* = 2.75, RMSEA = 0.067, IFI = 0.92, TLI = 0.91, CFI = 0.92)^20^.The Child Neglect Scale (partial) measures the extent of neglect experienced by the child. It consists of 29 items organized into three subscales: physical neglect, affection neglect, and communication neglect. Caregivers provided responses for each subscale. Higher scores on each subscale indicate greater levels of neglect in the corresponding dimension. The scale was developed for the Chinese cultural context with an internal consistency reliability of 0.848, a split-half reliability of 0.810, and a test-retest reliability of 0.897. The construct validity is good (*χ*^2^/*df* = 1.766, RMSEA = 0.047, GFI = 0.917, TLI = 0.916)^21^.The Brief Coparenting Relationship Scale (Brief CRS) evaluates the quality of coparenting in families. It assesses the level of support and cooperation between fathers and mothers in parenting, to evaluate the quality of the family environment. The mother completes the scale, rating to 14 items on a scale ranging from 1 (not very true) to 5 (very true). Higher scores indicate higher levels of parental support and cooperation in coparenting. The scale has demonstrated good reliability(0.613) and validity(factor analysis yielded five factors explaining a total of 65.780% of the variance in the full scale, with factor loadings greater than 0.4 for each item) in Chinese studies^22^. Finally, the Parenting Sense of Competence Scale (PSOC) was used to assess maternal self-efficacy in parenting. It consists of 17 items with scores ranging from 1 (strongly disagree) to 6 (strongly agree). The scale is divided into a performance subscale and a satisfaction subscale. A higher overall score reflects a greater sense of efficacy in parenting. In China, the scale has exhibited good reliability, with internal consistency reliability values of 0.82, 0.80, and 0.85 for the total scale and efficacy and satisfaction subscales, respectively. Additionally, it has good construct validity (*χ*^2^/*df* = 1.67, RMSEA = 0.059, NFI = 0.91, INNF = 0.92, CFI = 0.91, and IFI = 0.94)^23^.

**Table 1:** The duration information of Assessments/Procedures in periCBD.

Participant	Assessments/Procedures	Assessment age	Method	Duration (mins)	Validated in China
Children	Wechsler Preschool and Primary Scale of Intelligence	3–6	1vs.1 Task	90	Yes^50^
	Wechsler Intelligence Scale for Children	7–10	1vs.1 Task	90	Yes^49^
	Movement Assessment Battery for Children Second Edition	3–10	1vs.1 Task	30	Yes^30^
	Pittsburgh Sleep Quality Index (PSQI)	5–10	Online Questionnaire	10	Yes^43^
	Parental Mediation Scale (Children version)	7–10	Online Questionnaire	5	No
	Smartphone Addiction Proneness Scale (Children version)	7–10	Online Questionnaire	5	Yes^47^
Caregiver	Learning Disabilities Screening Scale (LDSS)	7–10	Online Questionnaire	5	Yes^36^
	Child Behaviour Checklist for Age 1^1/2^-5 (CBCL1^1/2^-5)	3–5	Paper Questionnaire	20	Yes^40^
	Child Behaviour Checklist 6-18-2001 version (CBCL6-18)	6–10	Paper Questionnaire	20	Yes^41^
	Developmental Coordination Disorder Questionnaire	3–10	Online Questionnaire	3	Yes^32^
	ADHD Rating Scale-Parent Version IV	3–10	Online Questionnaire	5	Yes^35^
	Autism Behavior Checklist	3–10	Online Questionnaire	10	Yes^38^
	The Life Event Scale (partial)	3–10	Online Questionnaire	3	Yes^20^
	Child Neglect Scale (partial)	3–10	Online Questionnaire	6	Yes^21^
	Parental Mediation Scale (partial) (Caregiver version)	3–10	Online Questionnaire	3	No
	Smartphone Addiction Proneness Scale	3–10	Online Questionnaire	3	Yes^47^
Mother	Self-rating Depression Scale	3–10	Online Questionnaire	5	Yes^17^
	Trait Depression Scale	3–10	Online Questionnaire	5	Yes^28^
	Self-rating Anxiety Scale	3–10	Online Questionnaire	5	Yes^17^
	Trait-Anxiety Inventory	3–10	Online Questionnaire	5	Yes^29^
	Edinburgh Postnatal Depression Scale	3–10	Online Questionnaire	3	Yes^27^
	Brief Coparenting Relationship Scale	3–10	Online Questionnaire	2	Yes^22^
	Parenting Sense of Competence Scale	3–10	Online Questionnaire	3	Yes^23^

#### Neurodevelopment conditions (NDCs)

The periCBD dataset also collects information about neurodevelopmental conditons (NDCs) and incorporates multiple questionnaires to assess these conditions. The details and detection rates of these assessments can be found in Table 2.

- **Motor Development** The Second Edition Movement Assessment Battery for Children (MABC-2) and the Chinese version of the Developmental Coordination Disorder Questionnaire (DCDQ-C) were used to evaluate the motor development of children. The MABC-2 is a widely-used standardized assessment tool for measuring children’s motor development. It is suitable for use in children aged 3–16 years and has three dimensions: manual dexterity, aiming and catching, and balance. Ke Li^30^established the norm for urban children in China, reporting an internal consistency reliability coefficient of 0.583, which is close to the acceptable range. CFI and TFI values of the scale are both approximately 0.9, and RMSEA is 0.045, indicating good structural validity^31^. The DCDQ-C, completed by the caregiver, is divided into three dimensions: control during movement, fine motor/handwriting, and general coordination. Zhu Qingqing and colleague^32^ applied it in Chinese children aged 4–6 years and found an internal consistency reliability coefficient of 0.867, indicating good reliability. The questionnaire also has good structural validity^33^.
- **ADHD Rating Scale-Parent version IV (ADHD RS-IV)** The assessment of ADHD were performed by ADHD Rating Scale-Parent version IV (ADHD RS-IV). The 18 ADHD RS-IV items are divided into the inattentive and hyperactive/impulsive dimensions. The results can be used to categorize individuals into four groups: no ADHD, ADHD predominantly inattentive type, ADHD predominantly hyperactive/impulsive type, and ADHD combined type. The Chinese version of the ADHD RS-IV has good internal consistency reliability, with a coefficient of 0.91. Additionally, it exhibits high construct validity (confirmatory factor analysis of the three-factor model: df = 132, *χ*^2^ = 905, RMSEA = 0.068, NFI = 0.93, NNFI = 0.91)^34,35^.
- **Learning Disabilities Screening Scale (LDSS)** The Learning Disabilities Screening Scale (LDSS) was used to investigate the specific learning disorder (SLD) among the school-age children. The LDSS has 18 items divided into 6 constructs: reading aloud, reading comprehension, spelling, written expression, calculation, and mathematical reasoning. It is suitable for the assessment of learning disabilities in Chinese children and has exhibited good internal consistency and sufficient structural validity and criterion correlation validity^36^.
- **Autism Behavior Checklist (ABC)** The Autism Behavior Checklist (ABC) was used to evaluate whether the children had autism spectrum disorder. This questionnaire, consisting of 57 items, is completed by the caregiver and serves to evaluate various characteristics associated with autism. The ABC items are divided into five subscales: (1) sensory; (2) relating; (3) body and object use; (4) language; and (5) social and self-help^37^.The checklist had a positive rate of 81.4% for clinically confirmed cases when applied to clinical samples in China^38^.

**Table 2:** MRI Protocols.

Site	Sequence	Time (min:sec)	TR (ms)	TE (ms)	Matrix	Flip Angle (°)	Resolution (mm)	Notes
AY	3D T1	4 m: 05 s	6.736	2.676	256 × 256	12	1.0 × 1.0 × 1.0	TI (ms) = 450
	3D T2	4 m: 36 s	3202	9.2036	256 × 256	90	1.0 × 1.0 × 1.0	
	rs-fMRI	8 m: 00 s	2000	30	64 × 64	90	3.9 × 3.9 × 4.3	
	DTI	7 m: 54 s	12800	78	112 × 112	90	2.0 × 2.0 × 2.0	
	pCASL	4 m: 14 s	4711	110	512 × 8	111	3.5 × 3.5 × 3.5	PLD = 1525 ms
LP	3D T1	6 m: 04 s	8.24	3.08	256 × 256	15	1.0 × 1.0 × 1.0	TI (ms) = 450
	3D T2	5 m: 59 s	1652	66.72	256 × 256	90	1.0 × 1.0 × 1.0	
	rs-fMRI	9 m: 02 s	2260	45	64 × 64	90	3.5 × 3.5 × 4.0	
	DTI	9 m: 03 s	15500	100	96 × 96	90	2.3 × 2.3 × 2.3	
	pCASL	4 m: 32 s	4690	108.12	512 × 8	155	3.5 × 3.5 × 3.5	PLD = 1525 ms

pCASL – PseudoContinuous Arterial Spin Labeling, TI - Inversion Time, PLD - post label delay.

#### Behavioural problems

- **Child Behaviour Checklist** Children’s behavioural problems were assessed by the Child Behaviour Checklist 6-18-2001 version (CBCL6–18) and Child Behaviour Checklist for Age 1^1/2^–5(CBCL1^1/2^–5)^39,40^. These questionnaires were completed by the caregiver. The CBCL1^1/2^–5 in China, demonstrating good validity (*χ*^2^ = 964.4, df = 416, RMSEA = 0.039, CFI = 0.920, TLI = 0.963)^40^. The CBCL6–18 was aslo utilized in a comparative cross-site study, investigating its application across different sites in China^41^.
- **Pittsburgh Sleep Quality Index** The Pittsburgh Sleep Quality Index was used to measure the participant’s sleep quality (in children 5–10 years old), A higher total score indicates poorer sleep quality^42^. In China, the index has good reliability(0.845) and good validity(*χ*^2^ = 53.16, df = 11, RMSEA = 0.09, CFI = 0.98, NNFI = 0.96, GFI = 0.97)^43^.
- **Parental Mediation Scale** The Parental Mediation Scale was adapted from the Television Mediation Scale, and was completed by the caregiver and by children aged 7–10 years. It consists of 7 items, with higher total scores indicating increased parental monitoring. The original Television Mediation Scale comprises 15 items and is divided into three dimensions: instructive mediation, restrictive mediation, and social coviewing. The scale was simplified focusing primarily on restrictive and co-viewing questions while also taking instructive dimensions into account. Consequently, the final selection included one question pertaining to instructive mediation, three questions concerning restrictive mediation, and three related to social co-viewing, totaling seven questions^44,45^.
- **Smartphone Addiction Proneness Scale** Caregivers and children aged 7–10 years completed the Smartphone Addiction Proneness Scale. This 15-item scale measures smartphone addiction across four subdomains: disturbance of adaptive functions, virtual life orientation, withdrawal, response, and tolerance. The scale has demonstrated good reliability within primary and secondary school populations^46^. Additionally, it has been found to have good reliability specifically within Chinese children and adolescents^47^.
- **Dictator Game** The dictator game was used to assess the sharing behaviour of children aged 3–6 years. This activity involves providing children with 30 stickers and requesting them to select their favourite 10 stickers. Once they have made their selection, they are informed that they have the option to give some of these 10 stickers to another child who will join later. It is emphasized to the children that they are not obligated to give away their stickers. They are then instructed to place the stickers they choose to keep for themselves in a brown envelope and the stickers they decide to give to others in a white envelope. The number of stickers given to others is recorded for analysis^48^.

#### Intelligence assessment

The Wechsler Preschool and Primary Scale of Intelligence-fourth edition (WPPSI-IV) and the Wechsler Intelligence Scale for Children-fourth edition (WISC-IV) were used to measure the intelligence of children aged 3–6 and 7–10 years, respectively^49^. The Chinese version of the WPPSI-IV, revised in 2014, is applicable to children aged between 2 years and 6 months and those aged 6 years and 11 months.For the Chinese sample, the reliability coefficients for each of the composite scores of the WPPSI-IV ranged from 0.85 to 0.94, and the full-scale IQ reliability was 0.96, with good validity^50^. The WPPSI-IV provides composite scores for full-scale IQ (FSIQ) and on 5 indices: the verbal comprehension index (VCI), visual spatial index (VSI) working memory index (WMI), fluid reasoning index (FRI), and processing speed index (PSI). In this study, measures of verbal comprehension, visuo-spatial skills and working memory were administered to children aged 3 years and 0 months to 3 years and 11 months. For children aged 4 years and 0 months to 6 years and 11 months, measures of fluid reasoning and processing speed were also included. The Chinese version of WISC-IV, revised in 2007, is suitable for children aged between 6 years and 0 months and 16 years and 11 months. The composite scores obtained from the WISC-IV include full-scale IQ (FSIQ) and 4 indice: the verbal comprehension index (VCI), perceptual reasoning index (PRI), working memory index (WMI) and processing speed index (PSI). For the Chinese sample, the mean reliability coefficients for each of the composite scores of the WISC-IV ranged from 0.87 to 0.97 and showed good validity^49^. The four index scales of the WISC-IV were utilized to measure the intelligence of children aged 7 years and 0 months to 10 years and 11 months in this study. All experimenters of the intelligence assessment for both WPPSI-IV and WPPSI-IV have undergone systematic training for measurement and have obtained the relevant certifications.

#### Cognitive Tasks/Tests

A battery comprising eight tasks was employed to evaluate children’s cognitive ability.

- **Dimensional Change Card Sort Task** This task was used to assess the cognitive flexibility of children aged 3 to 6 years. In this task^51^, children are instructed to first sort a set of cards (e.g., red rabbits and blue boats) in Phase 1 based on a preconversion rule. They are then instructed to resort the same set of cards in Phase 2 based on a postconversion rule. In Phase 3, participants are presented with a new set of cards (e.g., green flowers and yellow cars) and are instructed to classify them based on the rules established in Phase 2. In Phase 4, they are again presented with new cards and asked to classify them based on the rules from Phase 1. For example, children may be instructed to initially sort the cards based on colour, followed by sorting them based on shape. Subsequently, they are instructed to sort new cards by shape again, and finally, by colour once more. The number of correctly classified cards are counted at each stage. A higher score indicates better cognitive flexibility in children. The test materials were presented using PowerPoint, and children simply pointed to the selected cards on the screen with their hands.
- **Flanker Task** This task was employed to investigate children’s inhibitory control ability^52^. In the task, a white fixation cross “ + ” in the centre of a black screen for 1000 ms. After a 500 ms interval, a white target stimulus was presented. The specific target stimulus varied depending on the age of the children (fish for children under 7 years old, arrows for children 7 years old and older). The target stimulus consisted of five arrows (or fish), and the orientation of the central arrow (fish) either matched or did not match the orientation of the arrows (or fish) on either side. The children were instructed to focus on the direction of the central arrow (or fish) while ignoring the direction of the arrows (fish) on both sides. They were then instructed to respond quickly and accurately by pressing the “F” key if the direction was to the left or the “J” key if the direction was to the right. The inhibitory control ability of the participants was measured as the difference in response time between the inconsistent condition and the consistent condition. A greater difference in response time indicated poorer inhibitory control ability.
- **Wisconsin Card Sorting Test** The Wisconsin Card Sorting Test was implemented to measure cognitive flexibility in children aged 7–10 years^53^. During the test, children were presented with a computer screen that displayed four templates at the top: 1 red triangle, 2 green pentagrams, 3 yellow crosses, and 4 blue circles. A response card with varying shapes (triangle, pentagram, cross, circle), colours (red, yellow green, blue), and numbers (1, 2, 3, 4) would then appear randomly in the centre of the screen. Children were instructed to classify the response cards based on the four stimulus card templates. The test did not explicitly reveal the classification rule to the children; instead, they were informed whether each choice was correct or incorrect. The WCST yields several evaluation indicators: (1) total number of responses, (2) number of completed classifications, (3) number of incorrect responses, and (4) number of responses required to complete the first classification. A lower total number of responses, a greater number of completed classifications, fewer number of incorrect responses, and a smaller number of responses required to complete the first classification indicated better cognitive flexibility in children.
- **Self-Ordered Pointing Task** The task was used to measure working memory span in children aged 3–6 years^23^. In this task, children were presented with a book containing pictures. Initially, they viewed a page with two pictures and selected one of them. Subsequently, the researchers turned to the next page, which displayed the same two pictures as before but in different positions. The children were then instructed to point to the picture they had not previously chosen. This process was repeated with each new page introducing a new image placed alongside the original two, which were arranged differently from the previous page. The children had to identify the image that had not been selected in each arrangement. This continued until the child made an incorrect selection twice in a row after the images were rearranged. At that point, the task was concluded, and the number of images presented before task termination determined the working memory span of the participant.
- **Attention Network Test (ANT)** The classic attention network test (ANT)^54^ was applied to assess the three attention networks: alerting, orienting and executive attention. Each trial commenced with a “ + ” symbol displayed at the centre of the screen, serving as a reminder for children to maintain their focus on the centre of the screen throughout the experiment. Subsequently, an asterisk “*” appeared as a cue in the central position. There were four types of cues: no clue, central clue, double clue (two clues appeared above and below the fixation point at the same time) and spatial clue (one clue appeared above or below the fixation point). Instead of arrows, images of cartoon fish were employed to indicate a direction (left or right) according to the direction in which the head of the centre “fish” pointed, as a preference for children. The children were instructed to determine the direction of the centre “fish’s” head (left or right) as quickly and accurately as possible. Response times and accuracy were recorded for each trial. The effect value of the alerting network was computed by subtracting the response time in the two-cue condition from the response time in the no-cue condition. The effect value of the orienting network was calculated as the difference response time in the spatial cue context and the central cue context. The effect value of the executive control network was determined by subtracting the response time under consistent conditions from the response time under inconsistent conditions.
- **Inter-temporal choice task** This task was designed to measure the inter-temporal decision-making ability in children^55,56^. During the task, children were presented with multiple choices in which they had to indicate their preference for receiving a smaller immediate reward (2, 4, or 6 gold coins) or a larger fixed reward (8 gold coins) after varying delays (5, 15, 25 or 40 seconds). For example, in some trials, the children had to decide between obtaining 6 gold coins instantly or waiting for 25 seconds to receive 8 gold coins. All participants underwent testing in the same pseudorandom order. Each immediate small reward was paired twice with each delayed large reward, resulting in a total of 24 choice trials. The options were visually represented by two planes displayed on a computer screen, each indicating a specific number of gold coins. The delay was conveyed through the altitude at which the aircraft was flying, where a higher altitude indicated a longer delay. The positioning of the delayed reward aircraft on the left and right sides was balanced throughout the experiment. Participants made their choice by pressing a corresponding button (right or left), and the chosen gold coins were then “dropped” into a basket at the bottom of the screen either immediately or after the specified delay. After each trial, the computer visually updated the total number of coins accumulated before proceeding to the next trial. The participants were informed about the total number of trials they would complete, but they were not explicitly informed about the duration of the delays. Instead, they learned the length of each type of delay during the practice task, allowing them to mentally associate each aircraft altitude with the corresponding delay time. Upon completion of the task, participants received the gold coins they had accumulated. The inter-temporal decision-making ability was evaluated by calculating the area under the curve (AUC), which determined the “indifference point” for each delay time. A larger AUC indicated a greater willingness to wait and a higher level of inter-temporal decision-making in children.
- **Children**’**s Gambling Task** Kerr and Zelazot^57^ adapted the Iowa gambling task to create a simplified gaming task for measuring hot executive function in children. This research method is considered complex but effective. The task involving two decks of cards, one with vertical bars and the other with polka dots on the front. Turning the cards over reveals a happy face or a sad face on the back. However, there is a difference between the decks. The cards with vertical bars mostly have a happy face on the back and occasionally have a sad face, while the cards with polka dots mostly have two happy faces on the back but sometimes have four, five, or six sad faces. In this task, a happy face represents win, and the number of happy faces represent the number of coins won. A sad face represents a loss, and the number of sad faces represent the number of coins lost. Coins could be exchanged for a candy. Participants can choose only one card per trial. The cards with vertical bars yield fewer coins each time (only one) but also lead to fewer coins lost on average (only one). In contrast, the cards with polka dots yield more coins each time (two) but also lead to many more on average (losses of four, five, or six). Therefore, choosing the cards with vertical bars is advantageous in the long run, while choosing the cards with polka dots is disadvantageous. In the experiment, the children are told to try to win as many coins as possible by the end of the “game” (which consists of 50 card picks, unknown to the children beforehand). The first 25 choices are interpreted as children’s initial experience with the two types of cards, while the next 25 choices are used to assess emotional decision-making. The key dependent variable in this experiment was the affective decision index, which was calculated by subtracting the number of unfavourable cards chosen from the number of favorable cards chosen in trials 26 to 50. A higher affective decision index indicates stronger affective decision-making abilities in the participant.
- **Cake Gambling Task** This task was used to measure children’s risky decision-making abilities and is one of the research methods used to evaluate hot executive function^58^. The task involves dividing a cake into six pieces, with four pieces of one flavour (chocolate or strawberry) and two pieces of the other flavour. On the left and right sides of the cake, there are squares of pink and brown displaying different amounts of gold coins. The children are instructed to press a button to choose which flavour to bet on. If they make the correct choice, they receive a reward, but if they lose, they receive nothing. Throughout the gambling process, the cake with more pieces (4 pieces) is considered the low-risk option, while the cake with fewer pieces (2 pieces) is considered the high-risk option. The reward for the high-risk option (token = 2/4/6/8) is always twice that for the low-risk option. Therefore, the expected value (EV), calculated as the probability multiplied by the reward magnitude, is equal for both options. The total risky decision index was determined by calculating the proportion of the risky option. A higher value indicates a greater inclination towards risk decision-making and poorer risky decision-making abilities.

### Magnetic resonance imaging (MRI)

All paediatric MRI scans were conducted at two different hospitals: the Henan Anyang Maternal and Child Health Care Hospital, using a 3.0 T GE SIGNA Pioneer scanner and the Peoples Hospital of Liangping District, Chongqing, using a 1.5 T GE SIGNA Creator scanner, and A 21-channel head coil was used for AY site and 8-channel head coil was used for LP site. During the whole collection process, special efforts were made to avoid conducting the scans during meal times. Details about the imaging protocols are presented in Table 3. During the scanning process, participants were instructed to close their eyes, remain still and not to fall asleep. Two 3D morphometric sequences (T1 and T2), a resting-state fMRI sequence, a diffusion tensor imaging (DTI) sequence and an arterial spin labelling (ASL) sequence were leveraged to collect the MRI data. Due to the limitations of the clinical scanners, no field maps information was collected on either scanner. The total scan time for each session was 28 minutes 49 seconds in AY and 34 minutes 40 seconds in LP. Prior to the commencement of scanning, the experimenter will provide the children with a brief explanation that vibrations will be produced during the scan. The children will be informed that they can terminate the scan at any moment by pressing an emergency ball if they experience any discomfort.Each participant was equipment with child-specific earplugs for hearing protection. During the scanning procedure, an engineer is responsible for conducting the magnetic resonance scanning, while another experimenter is responsible for reading the instructions and monitoring the scan procedures and a parent or one of the experimenters will remain in the scanning room to accompany the child throughout the entire session. The children were instructed to close their eyes and stay as motionless as possible. All the scans are conducted solely for research purposes. In the event of an incidental finding in the structural image, parents or guardians will be immediately notified.

- **Morphometric MRI** Morphometric imaging consisted of a 3D T1 and a 3D T2 scan at both the AY and LP sites. The T1 images were collected with 1 mm isotropic voxels. The T1 sequence lasted for 4 minutes and 5 seconds in the SIGNA Pioneer scanner, while it took 6 minutes and 04 seconds in the SIGNA Creator scanner. For the 3D T2 image, a slice thickness of 1 mm was used. The acquisition time for this sequence was 4 minutes and 36 seconds on the SIGNA Pioneer scanner, and 5 minutes and 59 seconds on the SIGNA Creator scanner.
- **Resting-state fMRI** During the resting state protocol, participants were instructed to close their eyes, remain still, and avoid engaging in any specific thoughts, while also striving to stay awake. Resting state sequences with an 8-minute duration were acquired using a 3.9 × 3.9 inplane resolution and a 0.4 mm gap at the AY site and a 9 minutes and 2 seconds duration with 3.5 × 3.5 inplane resolution and 0.5 mm gap at the LP site.
- **Structural DTI** DTI scans on the 3.0 T Pioneer scanner consisted of 32 diffusion directions with a b-value equal to 1000 s/mm and 4 b-0 images, with 2 mm isotropic voxels, data collected in the AP direction. The acquisition time was 7 minutes and 54 seconds.DTI scans on the 1.5 T SIGNA Creator scanner consisted of 15 diffusion directions with a b-value equal to 1000 s/mm2 and 2 b-0 images, with 2.3 mm isotropic voxels. The acquisition time of data collection in the PA axis was 9 minutes and 3 seconds.
- **ASL MRI** The final sequence in each session was a pseudo-continuous arterial spin labelling (pCASL) sequence. The scan took 4 minutes and 14 seconds in the AY site, with a 3.5 mm resolution, and took 4 minutes and 32 seconds in the LP site, with a 3.5 mm resolution.

**Table 3:** Detail information NDCs Assessments.

Instrument	Disorder	Method	Participant	Age	Disorder Detection Rate		
					Total	NC	PF
Movement Assessment Battery for Children Second Edition (MABC-2)	DCD	Standardized Task	Children	3–10	32.4%	33.3%	29.9%
Developmental Coordination Disorder Questionnaire Chinese Version (DCDQ-C)	DCD	Online Questionnaire	Caregiver	3–10	24.7%	27.3%	17.4%
ADHD Rating Scale-Parent Version IV (ADHD RS-IV)	ADHD	Online Questionnaire	Caregiver	3–10	17.0%	19.7%	10.3%
Learning Disabilities Screening Scale (LDSS)	SLD*	Online Questionnaire	Caregiver	6–12	25.8%	27.5%	21.4%
Autism Behavior Checklist (ABC)	ASD	Online Questionnaire	Caregiver	3–10	1.2%	1.2%	1.5%

*Children who detect any disorder in each constructs of the Learning Disabilities Screening Scale are recorded as SLD.

## Data Records

### Data organization

In this dataset, the data file for each participant consisted of various imaging modalities, including structural imaging (T1 and T2 weighted images), resting-state functional MRI (rs-fMRI), diffusion tensor imaging and arterial spin labelling (ASL) perfusion imaging. The original data for each participant was converted into the NIFTI format and organized following the brain imaging data structure (BIDS) standard^59^. To ensure privacy, the facial information in each structural MRI was anonymized using the FaceMasking kit^60^, which obscures or removes identifiable facial features from the images.

### Data acquisition

The corresponding behavioral data have been made available and are accessible through ScienceDB^61^ with the following DOI: (10.57760/sciencedb.j00001.00423), Users will be granted access to the behavioral data upon completing the registration process and agreeing to the Data Usage Agreement of ScienceDB. All behaviour data are stored independently according to the participant ID. Each participant’s data comprise 10 files containing information on a total of 244 variables. These variables encompass a wide range of information, including demographics characteristics, cognitive abilities, intelligence measures, motor development assessments, disorder detection rates, parental behaviour, behavioural patterns, perinatal risk factors, and maternal emotional disorders.The neuroimaging data have been deposited into the Science Data Bank and are accessible through the “perinatal factors in child brain-mind development, periCBD” dataset^62^ (10.57760/sciencedb.10690) at Chinese Colour Nest Project (CCNP) – Lifespan Brain-Mind Development Data Community. This resource is also part of the Interdisciplinary Brain Database for *In-vivo* Population Imaging (ID-BRAIN) from the National Basic Science Data Center. Access to the data is granted upon requests submitted in accordance with the instructions described below. Samples of the neuroimaging data of both AY and LP sites are fully accessible through FigShare (10.6084/m9.figshare.25368610)^63^ to demonstrate all the details of the neuroimage data.

### Data license

To access the brain imaging data^64^, investigators are required to complete the Chinese Color Nest Project Data Use Agreements at 10.57760/sciencedb.o00133.00020 and submit it for review and approval by the Chinese Color Nest Consortium (CCNC). Requests for additional information and collaboration are welcomed and will be considered by CCNC; please review the Data Access Request and contact us via deepneuro@bnu.edu.cn

## Technical Validation

### Sample composition

#### Geographic distribution

The periCBD database presently encompasses data collected from two distinct sites: Anyang, situated in the northern region of Henan Province in central China, and Liangping, located in the northeastern part of Chongqing in China’s southwestern area. Chi-square analysis of gender distribution across these sites revealed no statistically significant differences (*p* = 0.3606). Furthermore, t-test also demonstrates that comparisons of age also failed to demonstrate any significant disparities (*p* = 0.548). Examination of total intelligence quotient (FSIQ) scores among children from both sites likewise indicated no significant variances (*p* = 0.5921). Results from the t-test also indicated that there was no significant difference in socioeconomic status (SES) between the Anyang (AY) and Liangping (LP) sites (*p* = 0.166). However, a significant finding emerged from the Chi-square analysis, highlighting that the Anyang site encompassed a greater prevalence of individuals with perinatal factors (*p* < 0.004).

#### Neurodevelopment conditions (NDCs)

In the preliminary phase of our investigation, we conducted an exploration into the incidence rates of Neurodevelopmental Conditions (NDCs) across the two study sites, AY and LP to assess whether there were significant differences in detection rates across several assessment tools, including the Movement Assessment Battery for Children-Second Edition (MABC-2), the Developmental Coordination Disorder Questionnaire-Children’s version (DCDQ-C), the Attention Deficit Hyperactivity Disorder Rating Scale-IV (ADHD RS-IV), the Learning Disability Screening Questionnaire (LDSS), and the Adaptive Behavior Composite (ABC). Utilizing chi-square analysis for statistical examination, we observed a notably higher detection rate of a specific neurodevelopmental disorder–Developmental Coordination Disorder (DCD)—at the LP site (MABC-2, LP: 42.5%, AY: 23.2%, *p* < 0.001). In contrast, for the detection rates measured by the other assessment scales, no significant differences were evidenced between the two sites.We also summarized the overall detection rates as well as the detection rates for the NC and PF groups for DCD, ADHD, learning disabilities, and autism in Table 2. Chi-square tests revealed that there are no significant differences in detection rates across any of the assessments of PF and NC groups.

#### Cognitive tasks

In the cognitive assessment section, we employed the flanker test and the Attention Network Test (ANT), both of which yield reaction time metrics, as our evaluative instruments. We established a threshold of 90% accuracy as the benchmark for satisfactory task completion; participants falling below this threshold were considered to have not adequately accomplished the task. Subsequent to our screening process, within the flanker test cohort, 27 children from the PF group met the benchmark, contrasted with 93 children from the NC group. Further statistical analysis using a two-way Analysis of Variance (ANOVA) with ‘group’ and ‘test conditions’ (congruent, incongruent) as factors revealed significant discrepancies solely in the test conditions(*p* < 0.001). Neither the ‘group’ condition (*p* = 0.968) nor the interaction between ‘group’ and ‘test conditions’ (*p* = 0.822) reached statistical significance. In the ANT, 27 children from the PF group and 82 from the NC group passed the benchmark. The results of the two-way ANOVA, taking ‘group’ and ‘network’ as factors, indicated a significant main effect for ‘network’ only (*p* < 0.001). Detailed distributions from both assessments are presented in Fig. 3.

### Quality assessment

In line with the protocols set forth by our previous endeavors in data collection and sharing, the PeriCBD mandates the availability of all imaging datasets to users irrespective of the data’s quality. This approach stems from the absence of a universally accepted standard delineating “high” from “low” quality data especially for children. Moreover, datasets which is ranked as of “lower quality” may serve as valuable resources in fostering the advancement of techniques for artifact.

#### Phenotypic data

**Fig. 2:**
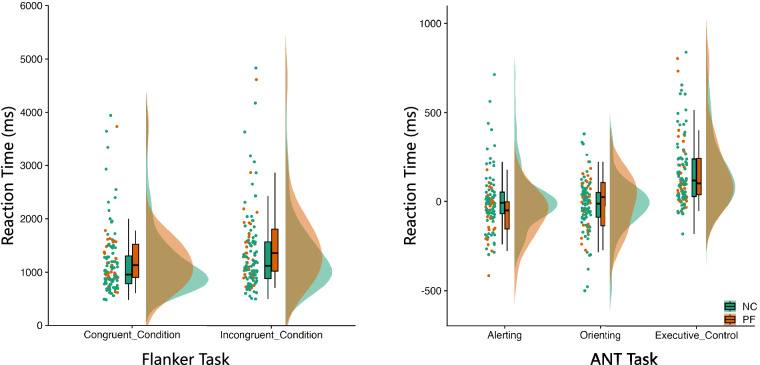
The reaction times for the flanker task and the Attention Network Test (ANT) task. The flanker task measured reaction times under congruent and incongruent conditions for both the PF NC groups. Meanwhile, the ANT task assessed the reaction times across three attention networks: the alerting network, the orienting network, and the executive control network, for both the PF and NC groups.

As shown in Fig. 2, all children were well clustered into the PF and NC groups based on their gestational age and birth weight. All the psychological and behavioural data are made available to database users, regardless of data quality. In addition, we have provided quality control (QC) files for each behavioural task and questionnaire, enabling users to make informed decisions about including specific data in their studies. The distributions of scores on the intelligence scale and the CBCL (both the social ability score and total score) are summarized in Fig. 2.

**Fig. 3:**
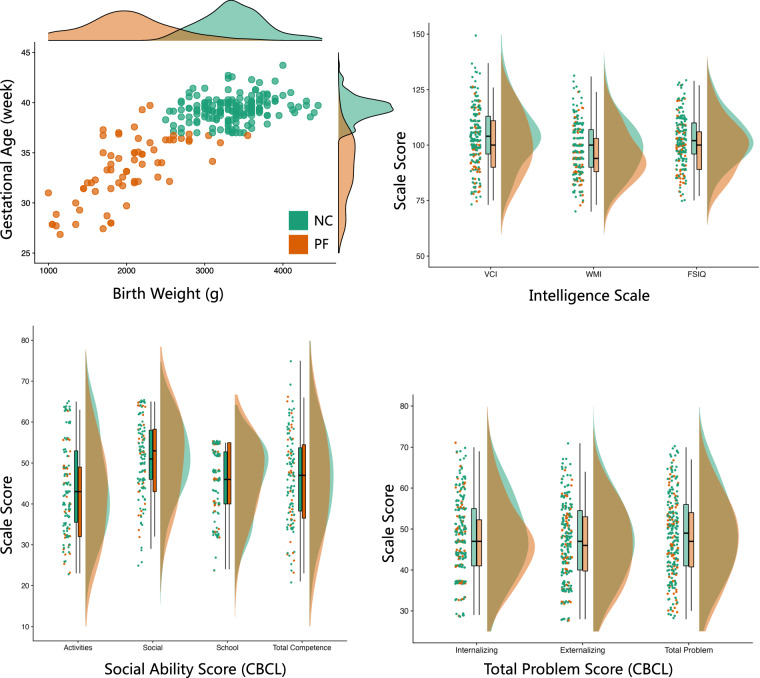
Distributions of non-neuroimaging measures. As depicted in the top-left panel, perinatal factors, including birth weight and gestational age differentiated children with perinatal factors (PF) from normal controls (NC). General intellectual ability (IQ) is visualized as (top -right) its standard score, including the verbal comprehension index (VCI), working memory index (WMI) and full-scale IQ (FSIQ). Mental health was evaluated by the Children Behaviour Check List (CBCL) in terms of social competence(bottom -left), including activities, social, and school competence, as well as total problems (bottom -right), including internalizing and externalizing problems.

#### Structural MR imaging

Among the 241 children, 184 (89 at the AY site, 95 at the LP site) underwent an MRI scan and were subsequently included in the quality control procedure. The MRI Quality Control tool (MRIQC), an automatic quality control pipeline, was utilized to obtain image quality metrics (IQMs) of T1 images such as the signal-to-noise ratio (SNR), coefficient of joint variation (CJV)^65^. Similar to previous studies^65,66^, manual rating procedures were conducted by two independent raters (coauthors: NJJ and LXL) who assessed all T1 images using an ordinal scale ranging from 0 to 2. They were aided by individual coronal T1-weighted images of the T1 visual reports generated from the MRIQC pipeline. In this rating framework, a rating of “0” indicated that the images were unusable and contained substantial artefacts. On the other hand, a rating of “2” indicated that the images were free from visible artefacts. Images with a rating of “1” had some artefacts present but were still considered usable. To assess the level of agreement between the raters, we utilized the percentage of agreement method, given by:

1 $$\frac{{q}_{11}+{q}_{22}+{q}_{33}}{\sum q}\times 100 \% $$

This equation calculates the sum of the diagonal element of the rating matrix *Q* and divides it by the sum of all elements of the rating matrix shown in Fig. 2. This percentage indicates the extent of agreement between the raters. In addition, weighted cohen’s kappa values (*κ*) were also calculated to further evaluate inter-rater agreement in terms of the initial visual ratings. The obtained results demonstrated a high degree of consistency between raters in evaluating T1 images at both sites. The inter-rater agreement was found to be 74.1% for the AY site and 79.6% for the LP site. Furthermore, the weighted Cohen’s kappa value was 0.796, with a 95% confidence interval ranging from 0.702 to 0.889 at the AY site. Similarly, at the LP site, the weighted Cohen’s kappa value was 0.838, with a confidence interval ranging from 0.758 to 0.919.

Based on the manual ratings, a total of 35 out of 89 images were excluded at the AY site and 26 out of 95 images were excluded at the LP site. Among the excluded images, 40% belonged to PF participants at the AY site, and 15% were from PF participants at the LP site (Fig. 2, middle row). Chi-square (*χ*^2^) goodness of fit tests were conducted to test whether the frequencies of images from PF and NC participants excluded via QC measures were significantly different from the frequencies in the original dataset. The results indicated that manual rating did not lead to a greater proportion of PF images excluded(AY site, *p* < 0.083, LP site, *p* < 0.48).

**Fig. 4:**
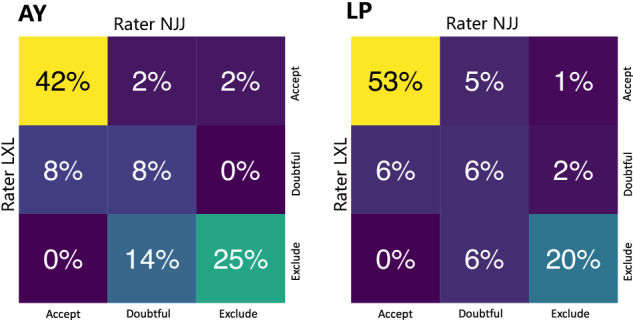
Summary of automatic and manual quality control of the images. this figure shows heatmaps of the overlap percent of quality labels assigned by rater NJJ and rater LXL for images from the AY and LP sites.

To investigate differences between the images labelled unusable and the included images in terms of head motion, we performed two-sample t tests on the coefficient of joint variation (CJV) of structural images The excluded images exhibited significantly higher CJV (*t* = 6.501, *p* = 7.44 × 10^−10^) than the included images (Fig. 4).

**Fig. 5:**
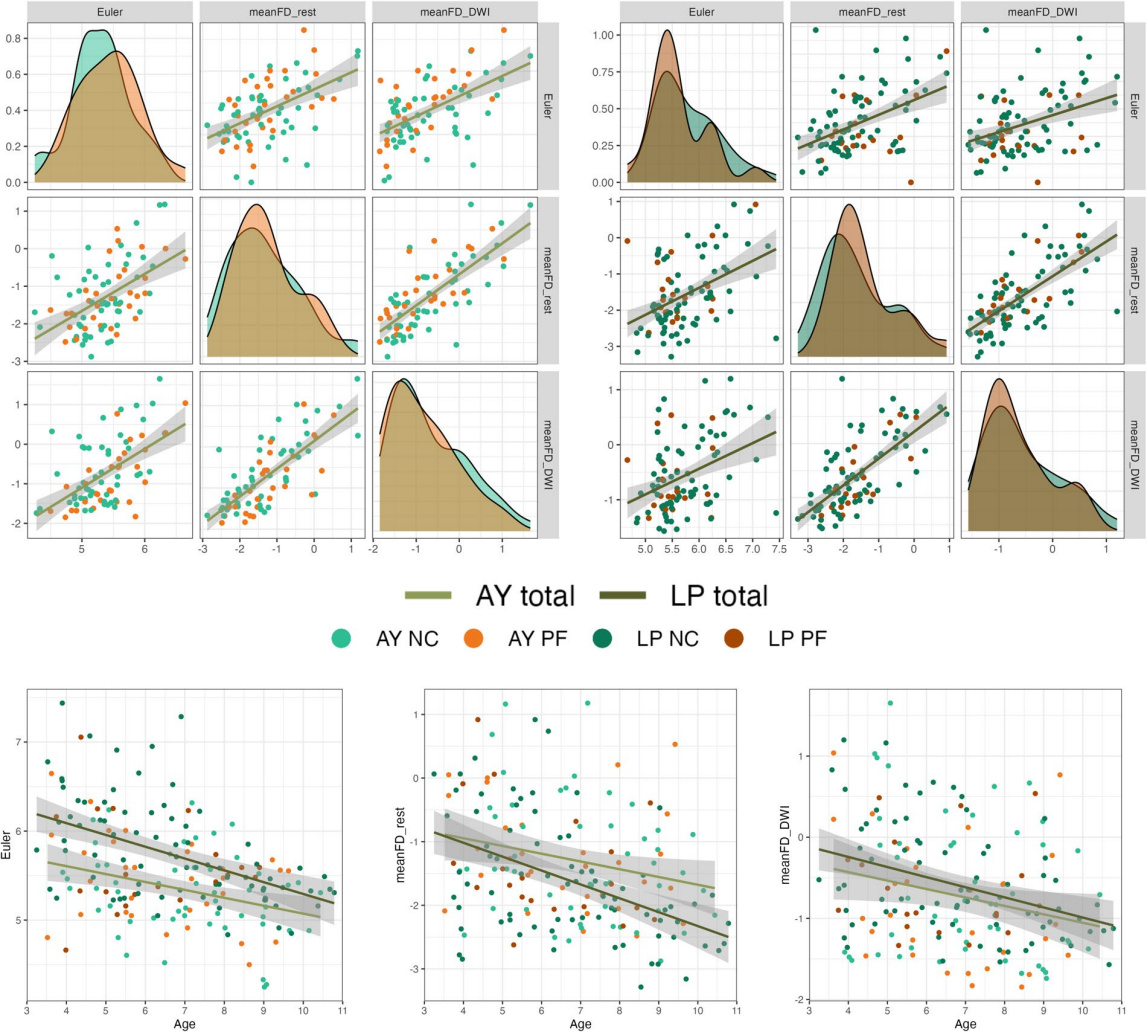
Image quality metrics (IQMs). The first panel illustrates the distribution and correlation of (IQMs) for T1 images (Euler number) and mean Framewise Displacement (FD) for resting-state fMRI (meanFD_rest) and Diffusion Weighted Imaging (meanFD_DWI) across both the Anyang (AY) and Liangping (LP) sites. The bottom panel elucidates the correlation between age and the IQMs for the AY and LP sites. The Euler number was transformed using a logarithm of the negative Euler number log(-1*Euler), while the other two metrics underwent a logarithmic transformation.

After two raters independently scored the images, the T1 images were excluded if at least one rater provided the “0” label. Figure 5 presents the proportion of the quality labels assigned by the two raters. The T1 images that passed QC were preprocessed using the Connectome Computation System (CCS)^67^ in the following steps: (1) Images were cropped with FSL’s **robustfov** to remove lower head and neck; (2) A spatially adaptive non-local means method was applied to denoise the images; (3) Skull stripping was performed using **deepbet**^68^, which involved training a new pediatric U-Net model based on the CCNP dataset^69^; and (4) FreeSurfer (version 6.0.0) was utilized to obtain morphological measurements of different brain morphometry^70^. During this step, the brainmask generated by FreeSurfer was replaced by the brainmask generated by **deepbet**. Eular number of each T1 image were derived by calculated the surface holes of both left and right hemispheres.

#### Resting-state functional MR imaging

The Image Quality Metrics (IQMs) for resting-state fMRI scans were extracted utilizing the MRIQC pipeline. Specifically, mean framewise displacement (meanFD) and temporal Signal-to-Noise Ratio (tSNR) were selected to assess the quality of resting-state images. The mean FD values for the PF group and the NC group were recorded at the AY site (PF: 0.404, NC: 0.459) and the LP site (PF: 0.410, NC: 0.332), respectively. Two-sample t-test indicate no differences on head motion between PF and NC groups on both sites.

In our latest study on head motion^71^, we introduced an age-specific criterion for data exclusion, anchored on the 93.5th percentile of the head motion growth curve, which equates to a mean FD of 0.2 mm in young adults. In this framework, we adopted two distinct threshold values for mean FD (0.3 mm for 6-year-old children and 0.5 mm for 10-year-old children, representative of the 93.5th percentiles of their respective head motion growth curves) serving as the criteria for our excluding participant due to head motion. When employing a mean FD of 0.3 mm as the exclusion criterion, 20 images from the NC group (38.5%) and 13 from the PF group (41.9%) were excluded in the AY site, whereas at the LP site, 21 images from the NC group (28%) and 6 from the PF group (30%) were omitted. When adopting 0.5 mm as the exclusion threshold,12 images from the NC group (23.1%) and 7 from the PF group (22.6%)were excluded in the AY site, whereas at the LP site, 16 images from the NC group (21.3%) and 5 from the PF group (25%) were omitted. Additionally, mean tSNR values for the PF and NC groups were calculated for both the AY and LP sites, revealing no statistically significant differences between groups (*p* < 0.26) or sites (*p* < 0.60), nor any significant interaction effects between group and site (*p* < 0.79).

#### Diffusion tensor MR imaging

The IQMs of diffusion MRI scans was calculated through the QSIprep pipeline^72^ Specifically, meanFD and the number of bad slices were selected to demonstrate the quality of diffusion images. The meanFD for the PF group and NC group were calculated at AY site (PF: 0.604, NC: 0.778) and the LP site (PF: 0.644, NC: 0.749). No significant meanFD difference was detected in both sites (AY: *p* < 0.307, LP: *p* < 0.427). In this section, we applied the 93.5th percentile of meanFD growth chart of head motion in 6-year-old children, which is 0.5 mm, as the criterion for head motion exclusion. At the AY site recorded exclusions of 19 individuals from the NC group (37.3%) and 13 from the PF group (43.3%). At the LP site, 35 participants from the NC group (47.3%) and 8 from the PF group (42.1%) were excluded. The mean number of bad slices was 1.57 in the PF group while 1.92 in the NC group at AY site, and was 0 in the NC group while 0.07 in the NC group at LP site.

#### Correlation between IQMs and age

Finally, we examined the distribution of the Euler Number (EN) for T1 images, as well as the Framewise Displacement for resting-state fMRI (rs-fMRI, meanFD_rest) and Diffusion Weighted Imaging (DWI, meanFD_DWI) at both AY and LP sites, their inter-correlations, and their correlations with age (Fig. 5). The results demonstrated that the quality control (QC) metrics for the three modalities showed high positive correlations at both sites (AY: EN - meanFD_rest *r* = 0.513, *p* < 0.001, EN - meanFD_DWI, *r* = 0.557, *p* < 0.001, meanFD_rest-meanFD_DWI *r* = 0.746, *p* < 0.001; LP: EN - meanFD_rest *r* = 0.448, *p* < 0.001, EN - meanFD_DWI, *r* = 0.380, *p* < 0.001, meanFD_rest-meanFD_DWI *r* = 0.690, *p* < 0.001). Simultaneously, all the three metrics exhibited negative correlations with age (AY: EN-age *r* = −0.335, *p* = 0.0017, meanFD_rest-age *r* = −0.244, *p* = 0.026, meanFD_DWI-age *r* = −0.225, *p* = 0.043; LP: EN-age *r* = −0.458, *p* < 0.001, meanFD_rest-age *r* = −0.455, *p* < 0.001, meanFD_DWI-age *r* = −0.364, *p* < 0.001).

## Usage Notes

All data acquired freely from the CCNC can be utilized solely for scientific research purposes. The use of image data requires users to fill out the corresponding DUA form^64^ at 10.57760/sciencedb.o00133.00020 and submit the form for review. To access behavioral data, users must register with Science Data Bank website and agree to all the DUAs on the website and download the data^62^ at 10.57760/sciencedb.10690. Users of this dataset are expected to acknowledge the contributions of the original authors and properly cite the dataset according to the guidelines provided on the Science Data Bank website (10.57760/sciencedb.10690). We encourage investigators to utilize this dataset in their publications, with the condition that this article is cited, and to contact us for further data sharing and collaboration opportunities.

### More perinatal factors

In this database, weight at birth and gestational age were utilized as the primary perinatal factors. Several other questionnaires were applied that captured perinatal factors across various dimensions, such as medication during pregnancy, exposure to toxic and harmful substances during pregnancy, gestational hypertension, gestational diabetes, pregnancy complicated with heart disease, cesarean delivery, birth defect, depression during pregnancy, anxiety during pregnancy, pressure during pregnancy, prenatal anxiety. By incorporating these data, researchers can employ a multifactorial analysis strategy to establish associations between brain development and the presence of perinatal factors.It is also important for users of the data to note that the measurements of these adverse perinatal factors were conducted at the time of the children’s participation in the tests, which is, the measurements were completed 3–10 years postpartum by the mothers. These factors are based on retrospective data, which may potentially affect the experimental results.

### Developmental coordination disorder

Developmental coordination disorder (DCD) is one of the most common NDCs among school-aged children and is characterized by delays in gross and fine motor development without apparent intellectual or medical causes. It is widely believed that DCD predicts the occurrence of various NDCs in 3 to 10 years old children. Furthermore, some researchers have proposed that DCD may serve as an early indicator of NDCs, considering that motor difficulties/disorders are commonly observed in all types of NDCs. In this dataset, we used the MABC-2 and the DCDQ-C to detect DCD. Researchers can further explore the relationship between NDCs and DCD by leveraging these data.

### Brain-wide association studies with perinatal factors

The effects of perinatal factors on the central nervous system have been widely acknowledged, with documented effects such as brain injury and atypical maturation following preterm birth^7^. These factors resulted in a high degree of heterogeneity in neurodevelopment due to substantial individual differences. Therefore, to elucidate the relationship between perinatal factors and neurodevelopment, it is important to establish brain-wide associations with perinatal factors at an individual level, referred to as individual BWAS-PF^73^. The advent of normative modeling provides an intuitive and effective approach to implement the individual BWAS-PF. By using growth charts derived from extensive data on brain development in healthy individuals, the relative positions of specific brain regions in terms of structural and functional metrics can be obtained, allowing the identification of deviation from the “normal” range^74^. This modeling approach also has promising applications in family intervention and clinical practice^75^. With advancements in normative model algorithms, these models can now be adjusted using out-of-sample MRI datasets with smaller sample sizes^76,77^. This enables the extraction of information on an individual’s relative position in terms of variables such as age, sex, and perinatal factors. Users can leverage this information to establish associations with perinatal factors and other behavioural metrics.

## Data Availability

No custom codes or algorithms were used in the generation or processing of data in this manuscript.
